# Multi-Layer Biosensor for Pre-Symptomatic Detection of *Puccinia strifformis*, the Causal Agent of Yellow Rust

**DOI:** 10.3390/bios12100829

**Published:** 2022-10-06

**Authors:** Mohamed H. Hassan, Abdalla M. Omar, Evangelos Daskalakis, Abubaker A. Mohamed, Lesley A. Boyd, Christopher Blanford, Bruce Grieve, Paulo JDS. Bartolo

**Affiliations:** 1 Department of Mechanical, Aerospace and Civil Engineering, University of Manchester, Manchester M13 9PL, UK; 2 Department of Materials, University of Manchester, Manchester M13 9PL, UK; 3 NIAB, Cambridge CB3 0LE, UK; 4 Department of Electrical & Electronic Engineering, University of Manchester, Manchester M13 9PL, UK; 5 Singapore 3D Printing Centre, Nanyang Technological University, Singapore 639798, Singapore

**Keywords:** 3D printing, bioinspired, *Puccinia striiformis*, yellow rust, biosensor

## Abstract

The yellow rust of wheat (caused by *Puccinia striiformis* f. sp. *tritici*) is a devastating fungal infection that is responsible for significant wheat yield losses. The main challenge with the detection of this disease is that it can only be visually detected on the leaf surface between 7 and 10 days after infection, and by this point, counter measures such as the use of fungicides are generally less effective. The hypothesis of this study is to develop and use a compact electrochemical-based biosensor for the early detection of *P*. *striiformis*, thus enabling fast countermeasures to be taken. The biosensor that was developed consists of three layers. The first layer mimics the wheat leaf surface morphology. The second layer consists of a sucrose/agar mixture that acts as a substrate and contains a wheat-derived terpene volatile organic compound that stimulates the germination and growth of the spores of the yellow rust pathogen *P. s.* f. sp. *tritici*. The third layer consists of a nonenzymatic glucose sensor that produces a signal once invertase is produced by *P. striiformis*, which comes into contact with the second layer, thereby converting sucrose to glucose. The results show the proof that this innovative biosensor can enable the detection of yellow rust spores in 72 h.

## 1. Introduction

To accommodate for the rapidly increasing global population, it is estimated that global food production would need to increase by 70% to avoid a food crisis occurring [1,2,3,4,5].

Wheat (*Triticum aestivum* L.) is an important cereal crop and a staple source of food for approximately 40% of the world’s population [6]. In 2021, the global production of wheat was 778.6 million metric tons, with the European Union, China, and India being the three largest producers of it [7]. Due to the importance of this crop, wheat yield losses present a major problem for global food security [8,9].

Wheat yellow rust fungi (*P. s.* f. sp. *tritici)* is a fungal, biotrophic pathogen. Yellow rust can affect the grain quality, thereby reducing the protein content, as well as reducing the yield [10,11]. As an air-borne pathogen, the spores can be transported considerable distances via the wind, and they can also be carried on animals and human clothing [12,13]. Yellow rust is found predominantly in temperate and maritime environments, and its optimal temperature for infection and growth ranges between 7 and 22 °C [14,15].

Current strategies for yellow rust management are based on the use of genetically resistant wheat varieties and fungicide applications as part of an Integrated Pest Management scheme [16,17]. However, this contributes to the presence of pesticide-resistant fungal strains [18,19]. In addition, the timing of the fungicide application is critical for the treatments to be effective

**Figure 1:**
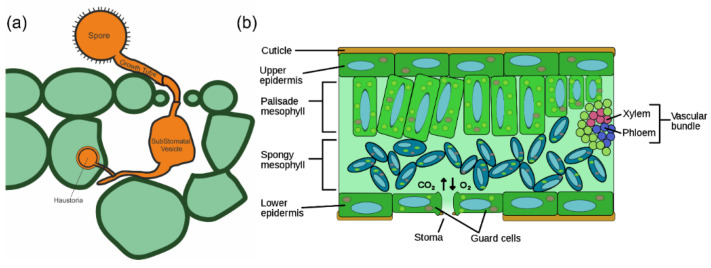
(**a**) Schematic representation of early infection structures of *Puccinia striiformis*. The spore germ tube enters the leaf through the stomata, thereby forming a substomatal vesicle from which infection-hyphae develop. Once the hyphae contact the mesophyll cell, a haustoria is formed inside the plant cell. (**b**) Transverse section of a plant leaf showing the different layers of the leaf.

*P. s.* f. sp. *tritici* spore germination can be seen within three hours of spores being deposited onto the leaf surface (Figure 1). The spore absorbs the free moisture on the leaf surface, thus producing a germ tube. The cytoplasm within the spore moves into the growing germ tube as it orientates itself to be perpendicular to the epidermal cells of the leaf surface until it reaches a stomata [20,21,22,23]. Between 6–8 h after the inoculation, the germ tube grows between the guard cells of a stomata and by 8–12 h, a substomatal vesicle is formed inside of the stomatal cavity. Primary infection hyphae develop on the substomatal vesicle after 12–18 h [23,24]. A number of studies have suggested that *P. s.* f. sp. *tritici* produces its own invertase as it is required to aid the nutrition uptake of the pathogen by converting sucrose to glucose and fructose [25,26]. Yellow rust also leads to an increase in the number of the reactive oxygen species that are in the infected plants, hence reducing the plant’s overall photosynthetic efficiency, thereby leading to yield loss [27].

**Figure 2:**
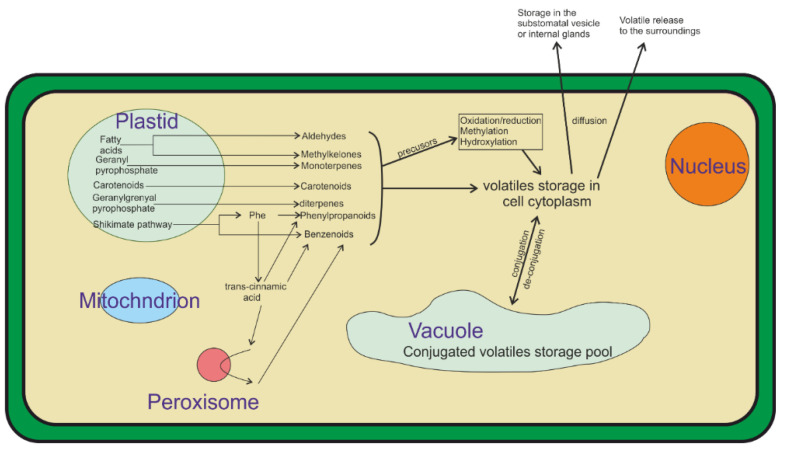
Most plant volatiles are derived from four biosynthetic classes: aromatics, fatty acid, terpenoids, and amino acids. Volatiles are produced from fatty acids via oxidative cleavage. Several of these products are modified to be more lipophilic prior to release through the masking or removing of hydrophilic functional groups through methylation or reduction reactions. Figure modified from [28].

The volatile organic compounds (VOC) are the metabolites that are produced by the plants that are released into the air. Plants use volatiles to protect themselves from abiotic and biotic stresses, and to supply information (or disinformation) to mutualists (ex: pollen carriers) and competitors [28,29]. The VOCs act as information transferors, resolving many problems that are faced by the plant’s lack of mobility. The VOC blends are dominated by four biosynthetic classes, terpenoids, fatty acids, amino acids, and compounds with aromatic rings (Figure 2). Several lipophilic volatiles are released from the membranes of the plant’s epidermal tissues. However, in the leaves and stems, the VOCs are released through the stomata, therefore the opening and closing of the stomata influences the volatiles’ release [28,29].

A major challenge that is related to yellow rust is that the disease’s symptoms can only be visually detected 7 to 10 days after the infection. However, at this stage, the use of preventative fungicides is largely redundant as the production of large numbers of spores are seen 14 days after the infection which spread the disease through the field. This raises the need for an early pathogen detection system that would enable farmers to apply preventative fungicide treatments well in advance of the infection and disease establishment stages.

Hyperspectral imaging technologies are used for plant disease detection procedures, and recently they have been increasingly investigated for yellow rust detection [30,31,32]. Spectral sensors measure the light that is reflected from the plant’s surface [33]. As the disease develops, the light spectrum shifts, thereby enabling the disease’s detection [34,35,36]. These systems use machine learning processes, and to operate, they properly require the crops to be monitored for a few seasons to collect enough data to enable that a subsequent accurate detection is made as well as to detect the symptomatic responses of the plants. Thus, their immediate deployment and use is not possible. Moreover, these systems are prone to errors due to different angles of sunlight being reflected from the plant’s surface.

Biosensors are used for agricultural and environmental monitoring applications to detect various pathogens and other environmental contaminants [37,38]. Due to their relatively high detection rate and short response time, the frequency of the use of biosensors is increasing [39,40]. However, the main challenges that is faced by current biosensors lie in their sensor performance, sampling, scaling up, and detection in open areas [37,38]. Roy et al. [41] fabricated an array of whole-cell biosensors to detect the aromatic pollutants such as benzene, phenols, and toluene, which are carcinogenic compounds that are found in polluted water sources. The sensor was able to detect the inert pollutants without any functional groups. However, this biosensor was temperature sensitive, which limited its operation in the field. Moreover, the authors did not provide any details regarding the detection time. Shi et al. [42] designed a colorimetric nanobiosensor to detect acetamiprid in soil. The sensor exhibited a high stability, sensitivity, and selectivity. However, it required that the soil samples be air-dried and that the ground be passed through a 1 mm sieve, then oven dried at 35 °C for 48 h. Then, dichloromethane (DCM) was added to the sample and filtered, and finally it was mixed with the sensing solution [42].

Advances in the field of nanomaterials have produced a promising nanoparticle-based biosensors. Nanoparticles are used for both enzymatic and non-enzymatic glucose biosensors, incorporating nanomaterials (e.g., noble and transition metal nanoparticles, CNTs, graphene, and nanostructured metal oxides) to amplify the electron transfer rate, thereby improving the biosensor’s performance in terms of its selectivity and sensitivity [43,44].

**Figure 3:**
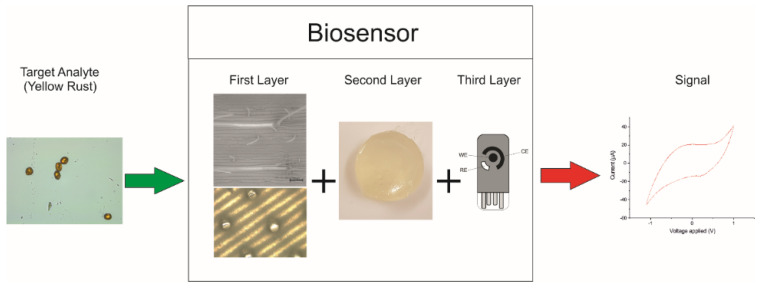
Operational rationale of proposed yellow rust biosensor. The signal can be obtained directly as a CV curve indicating the presence of glucose and therefore yellow rust.

The research that is presented here proposes an innovative, pre-symptomatic yellow rust detection system that consists of a three-layered biosensor, thus representing the first biosensor for yellow rust detection. The proposed biosensor has a low cost and it is accessible, in-field detection device for yellow rust. The material and fabrication costs have been kept below £5 per unit. The production of the sensor is a simple and direct method that used commonly found apparatus and has an average lead time of 15 min. The operational rationale of the proposed modular sensor is shown in Figure 3. The target analyte (spores of *P. s.* f. sp. *tritici*) lands on the artificial surface of the sensor (first layer). This layer mimics the wheat leaf morphology with parallel venation and artificial stomatal openings. Following the spore’s landing, germination takes place. The spores produce germ tubes that grow through the artificial stomatal opening towards the second layer, which consists of a sucrose/agar mixture that acts as a feeding media, and a plant VOC that acts as a growth-enhancing stimulant [29]. Invertase is produced by the germinated spores, thus breaking down the sucrose into glucose. The third layer is a nonenzymatic glucose sensor that detects the glucose [26], generating an electrochemical response which can be detected using cyclic voltammetry (CV), thereby signalling the presence of the target pathogen.

## 2. Materials and Methods

### 2.1. Materials

Polyethylene terephthalate glycol (PETG) was purchased from RS components (Northants, UK). Micrux single thick screen-printed electrodes were purchased from Alvatek,(WE: Carbon/RE: Silver/CE: Carbon) (Hampshire, UK). Gold (III) chloride (AuCl_3_), nickel acetate tetrahydrate (Ni (OCOCH_3_)_2_ · 4H_2_O), N, N-dimethylformamide (DMF), activated carbon (AC), dichloromethane (DCM), ethanol, agar A4550, dimethylamine borane (DMAB), terpene, and sucrose were purchased from Merck (Dorset, UK).

### 2.2. Sensor Fabrication

**Figure 4:**
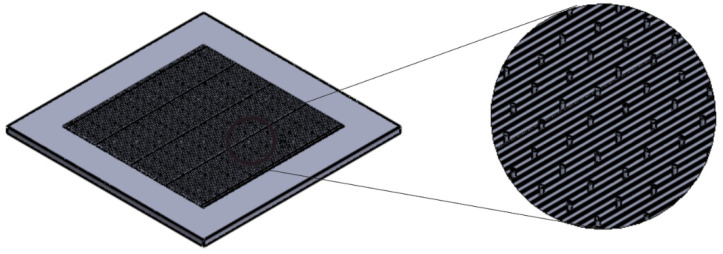
3D model of the designed biomimetic leaf surface.

**Figure 5:**
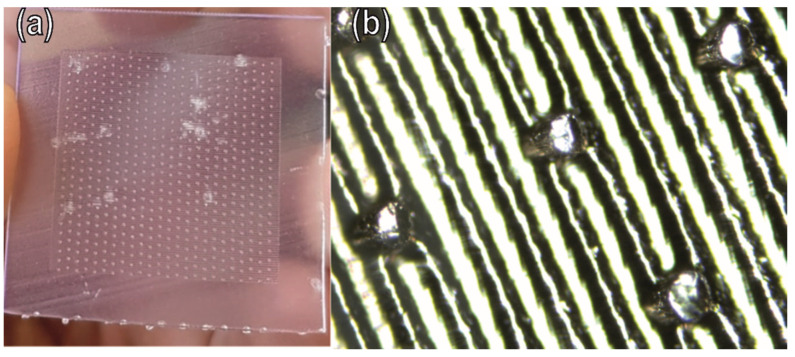
(**a**) Printed mould; (**b**) optical microscope image presenting the details of the printed model.

The sensor biomimetic surface was fabricated through a casting process. A mould was previously designed using Solidworks (Dassault systems, Waltham, MA, USA), mimicking the wheat leaf surface (Figure 4). Formlabs Form3 (Formlabs, Somerville, MA, USA) was used to produce the mould with a resolution of 100 µm (Table 1). After printing the mould (Figure 5), a PETG solution was prepared by dissolving PETG in DCM 1:10 ratio (*w*/*v*). The PETG solution (0.005 pas) was then poured into the mould and left to dry for two hours. The produced layer was then peeled off.

The second layer was fabricated by mixing Agar A4550 and sucrose in a 1:4 (*w*/*w*) ratio. Briefly, 0.1 g of agar and 0.4 g of sucrose were added to 20 mL of de-ionised water and stirred until a homogenous solution was obtained. The solution was heated up to 100 °C using a hot plate, cooled down to 35 °C to allow for gelation, and left to cool down to room temperature. Finally, a terpene VOC was injected into this agar gel.

**Figure 6:**
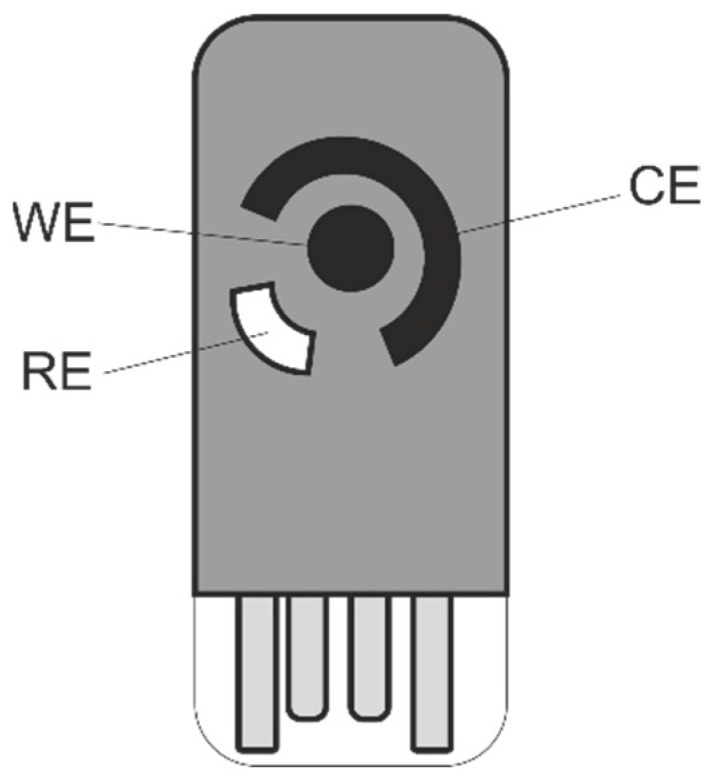
Schematic of the screen-printed electrode. WE: working electrode, CE: counter electrode, RE: reference electrode.

The third layer consists of an Au-Ni/AC-modified screen-printed electrode. An equal amount of gold (III) chloride and nickel (II) acetate (0.25 mmol) was dissolved in 15 mL of ethanol, which was followed by the addition of 2.5 mmol of activated carbon. Activated carbon was used to increase the surface area to amplify the sensing response by having a larger area for interaction between the glucose and the nanoparticles. The solution was stirred until a homogenous mixture was obtained and refluxed for two hours at 90 °C. Then, dimethylamine borane (DMAB) was added (1 mg), and the mixture was refluxed again for an additional hour. DMAB was used for the reflux, and this is used for activation of the gold-nickel-activated carbon nanocomposite. Finally, the obtained mixture was dried to obtain the sensing gold-nickel-activated carbon nanocomposite (Au-Ni/AC). The screen-printed electrode was modified using drop casting. Briefly, 1:10 (*w*/*v*) of Au-Ni/AC was added to DMF. The solution was sonicated for 30 min ensuring that the material was completely dispersed. Finally, 10 µL of the solution was drop casted on the SPE working electrode (WE) (Figure 6). The SPE was left to dry for 24 h in the fume cupboard.

### 2.3. Inoculation

The bioactivity of the top layer of the biosensor was tested by inoculating with *P. s.* f. sp. *tritici* spores. Briefly, the surface was placed in the middle of a stainless-steel pipe that was covered by an aluminium sheet at the top and bottom (spore inoculation tower). A small hole at the side of the steel pipe was used to blow the spores into the pipe, with the spores settling onto the biosensor surface by gravity. The surfaces were left in the steel pipe for 15 to 30 min to ensure that all spores had settled to the bottom of the pipe. The inoculated surfaces were then inspected under a light microscope to confirm that the spores had landed on the surface. The inoculated biosensors were placed in an incubator at 7 °C in total darkness at 60%humidity for 24 h.

### 2.4. Morphology

*P. s.* f. sp. *tritici* spore growth was investigated using optical microscopy (Keyence VHX-5000; Keyence, Milton Keynes, UK). The images were obtained using a top light at two magnifications (×5 and ×10). The light exposure was 100%.

The morphology of both the first and third layers of the biosensor was characterised using the scanning electron microscopy (SEM) Tescan Mira3 system (Tescan, Kohoutovice, Czech Republic). The first layer was platinum coated prior to imaging using the 108-auto sputter coater (Cressington scientific instruments, Watford, UK). Imaging was performed using 20 KV acceleration voltage. Images were analysed using ImageJ (Laboratory for Optical and Computational Instrumentation, University of Wisconsin, Madison, WI, USA) to determine the pore size of the first layer and the gold-nickel dispersion on the surface of the third layer. The morphology of the third layer was further studied using atomic force microscopy (AFM) nanosurf FlexAFM system (Nanosurf AG, Liestal, Switzerland). The structure was assessed using X-ray diffraction (XRD) which was carried out using X^’^Pert Pro X^’^celerator (Malvern Panalytical, Malvern, UK). Spectra were collected in the range of 2θ between 20° and 100°.

### 2.5. Electrochemical Measurements

The electrochemical behaviour of the sensor was assessed through cyclic voltammetry, and chronoamperometry using the µAUTOLABIII/FRA2 potentiostat (Metrohm Autolab, Utrecht, The Netherlands), and measurements were conducted at room temperature using a lab-made electrochemical cell. Cyclic voltammetry was performed using a potential range −1.0 V to 1.0 V with a scan rate of 100 mVs^−1^ to monitor the onset-potential of the prepared sensors towards the glucose catalytic oxidation from glucose to gluconolactone. Chronoamperometry was performed using a potential of 0.66 V over a time of 600 s.

Electrochemical Impedance Spectroscopy (EIS) was used to evaluate the performance of the sensor using the µAUTOLABIII/FRA2 potentiostat (Metrohm Autolab, Utrecht, The Netherlands). This technique applied an alternate current (AC) rather than a continuous one (DC). To obtain the optimal frequency, a frequency range of 100 kHz–0.1 Hz was used for impedance spectroscopic measurements. The amplitude of oscillation (AC) was set to 10 mV_RMS_. The optimal working potential was set to +0.66 V for measurements of glucose, respectively.

## 3. Results and Discussion

### 3.1. Morphological Characterisation

**Figure 7:**
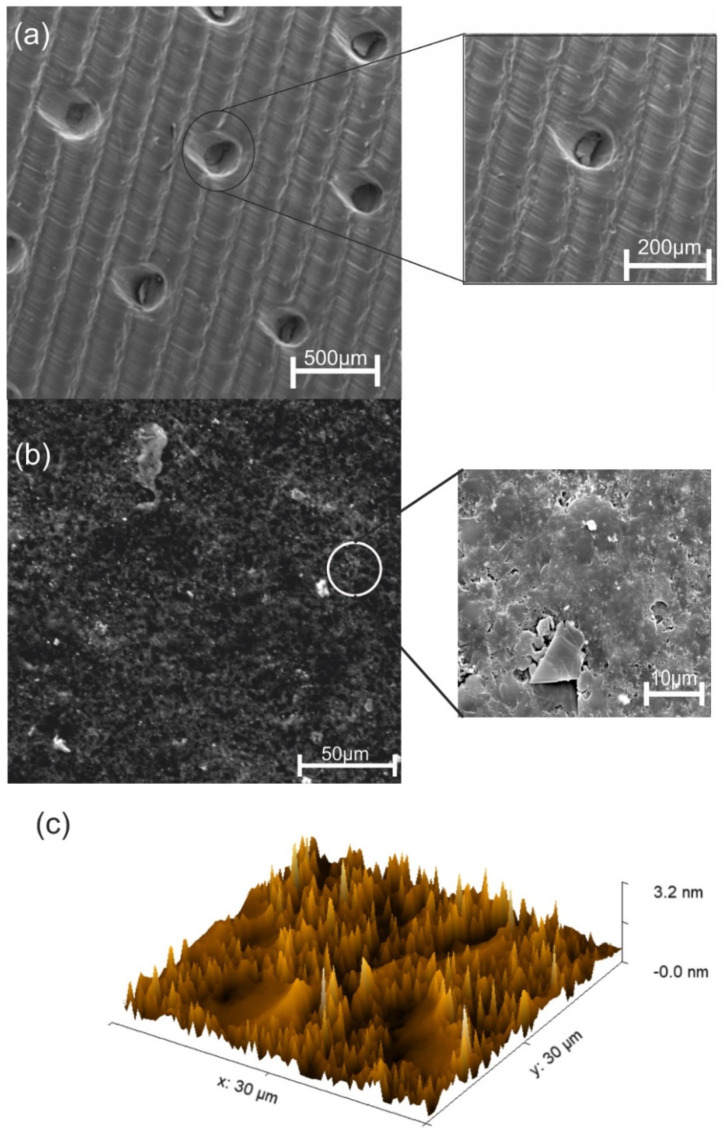
(**a**) SEM image of the casted biomimetic PETG layer. The created parallel venation mimics the wheat leaf with a width of 200 µm. The detailed, zoomed SEM image shows a stomatal hole with a diameter of 50 µm with an opening of 15 µm. (**b**) SEM images of the Au-Ni/AC modified screen-printed electrode, presenting two distinct regions. The zoomed-in SEM image clarifies the two regions where the carbon bed presents a nano-porous structure, thus allowing glucose to flow, and the top region, which is a cotton-like nanostructure consisting of Au-Ni particles. (**c**) AFM image of the Au-Ni/AC modified screen-printed electrode exhibiting cylindrical round cap columns with channels allowing glucose to pass through.

Read et al. [45] investigated the growth of *Puccinia graminis* f. sp. *tritici* (wheat stem rust) and *Puccinia hordei* (barley brown rust) on the artificial surfaces that mimic the leaf morphology using polyesters. As *P. striiformis* is related to *P.g.* f. sp. *tritici* and *P. hordei*, it was assumed that *P. striiformis* would be able to grow on similar materials. Therefore, it was decided that they would use a material with the same backbone, polyethylene terephthalate glycol (PETG), and that they would fabricate the biosensor by 3D printing. PETG is a bioactive and biocompatible material [46].The moulded PETG layer (first layer of the biosensor) was designed to mimic the wheat leaf surface morphology, presenting parallel grooves and stomatal holes (Figure 7a). The parallel grooves had a size of 200 µm, and the stomatal holes an average size of 50 µm, with openings with an average size of 15 µm. The SEM images of the Au-Ni/AC modified screen-printed electrode captured two distinct regions (Figure 7b). The upper region exhibited a cotton-like nanostructure, while the bottom region showed a brain-like nano porous structure with small grains and channels. From the SEM images it was also possible to observe the presence of gold and nickel (light white) and activated carbon (black). The atomic force microscopy (AFM) results of the third layer (Figure 7c) showed that there was the presence of round cap columns that were composed of several grains ranging between 40 nm to 1 µm. Channels with an average width of 5 µm were formed around the columns, thus allowing the glucose to pass through them.

### 3.2. Electrochemical Behaviour of the Au-Ni/AC SPE

**Figure 8:**
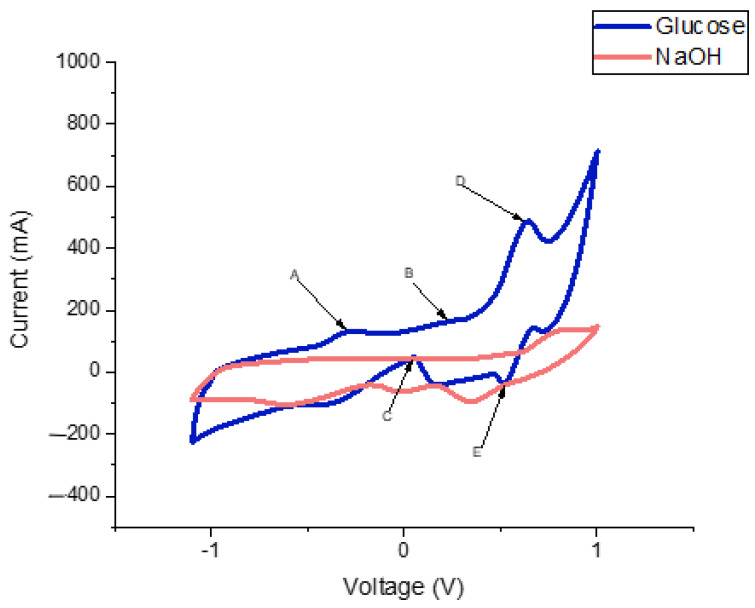
CV curve of the Au-Ni/AC modified screen-printed electrode using NaOH as a buffer. The red curve corresponds to NaOH without glucose. The blue curve corresponds to NaOH in the presence of glucose. From the blue curve it is possible to observe the oxidation peaks at peak A (−0.27 V), peak B (0.22 V), peak E (0.53 V), and peak D (0.66 V), and a reduction peak at peak C (0.04 V).

The electrochemical behaviour of the Au-Ni/AC SPE toward glucose oxidation was assessed to evaluate its potential for glucose sensing. Figure 8 presents the CV curve of the Au-Ni/AC SPE in the absence (blue) and presence (red) of 10mmol of glucose in 0.1 mol NaOH. The CV curves that were obtained from the sensor exhibit four oxidation peaks at −0.27 V (peak A), 0.22 V (peak B), 0.53 V (peak E), and 0.66 V (peak D), and one reduction peak at −0.04 V (peak C). Peaks A, B, and C correspond to the typical two-step Au-glucose oxidation process, whereas peak C represents the reduction step that occurs in order to regenerate the sensor active sites, despite the presence of Ni. The electrocatalytic mechanism of Au-Ni/AC for glucose is a multistep process [47]. The process is initiated when the glucose molecules are dehydrogenated and adsorbed onto the surface of the Au-Ni/AC particles, which is followed by an increase in the metal-OH_ads_ that increases the potential, hence mediating the catalytic oxidation of the intermediaries towards gluconolactone.

Similar to glucose oxidation that occurs on pure gold surfaces [48,49], the glucose oxidation that occurs on the surface of the Au-Ni/AC SPE mainly depends on the quantity of the metal-OH_ads_ as well as on the premonolayer oxidation of the metal-producing metal-OH_ads_. Peak A (Figure 8) can be linked to the glucose dehydrogenation-forming adsorbed intermediate products. The intermediate accumulation that is due to the limited number of metal-OH_ads_ sites is produced at a lower potential (−0.27 V), blocking the Au-Ni/AC SPE surface active sites, and hence, decreasing the current. Peak B at 0.22 V is attributed to the consecutive catalytic oxidation of the adsorbed intermediates due to the increased metal-OH_ads_ sites, while peak D corresponds to the formation of metal oxides. In the negative potential scan, there is a current increase at 0.04 V that is due to the reduction of surface metal oxides that have a more negative potential than 0.23 V, and the presence of enough metal-OH_ads_ sites for the glucose catalytic oxidation. A similar behaviour was observed by Gao’s group, using a different setup, where Au-Ni were used as a core and shell, respectively, and this showed an increase in the anodic current at a potential of 0.26 V [50].

Peaks D and E are also associated with the conversion between Ni(II) and Ni(III) as they share a CV behaviour that is similar to that of Ni-based electrodes [51]. The Ni reaction mechanism in the alkaline medium for glucose oxidation can be explained as follows:

Ni + 2OH^−^ → Ni (OH)_2_ + 2e^−^ (1)

Ni (OH)_2_ + OH^−^ → NiO (OH) + H_2_O + e^−^ (2)

NiO (OH) + glucose → glucolactone + Ni (OH)_2_ (3)

**Figure 9:**
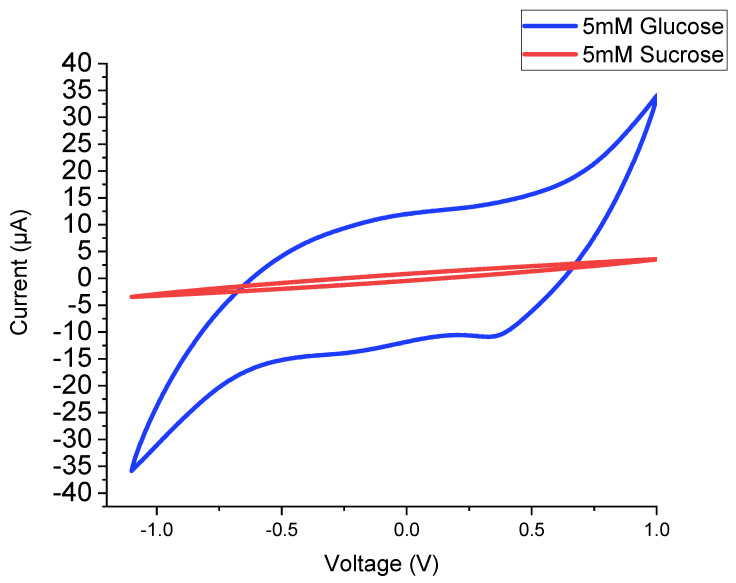
CV curves without a buffer (NaOH). The blue curve represents the response to 5 mM glucose. The red curve represents the response to 5 mM sucrose.

The sensor was then tested for its potential to perform glucose detection without a buffer to simplify the sensor. As it can be observed from the CV profiles (Figure 9), it is possible to differentiate between the different electrochemical responses of glucose and sucrose.

**Figure 10:**
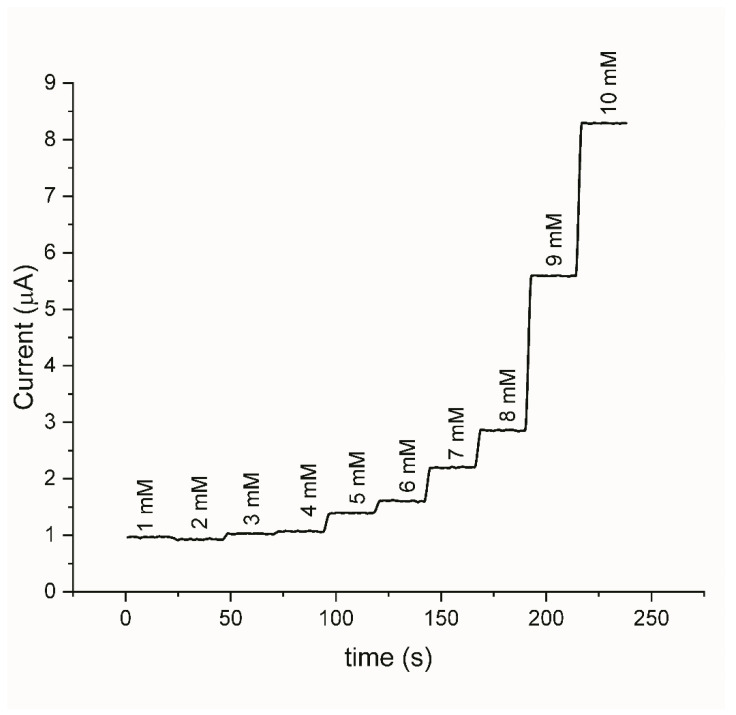
Current–time responses at +0.66 V with an increasing glucose concentration.

The amperometric response of the Au-Ni/AC SPE was investigated at 0.66 V (Figure 10), which corresponds to the oxidation peak that was observed on the CV. The glucose was successively added into the 0.1 mM of NaOH at 24 s intervals for 20 steps. The sensor presented with an extremely fast and stable response.

**Figure 11:**
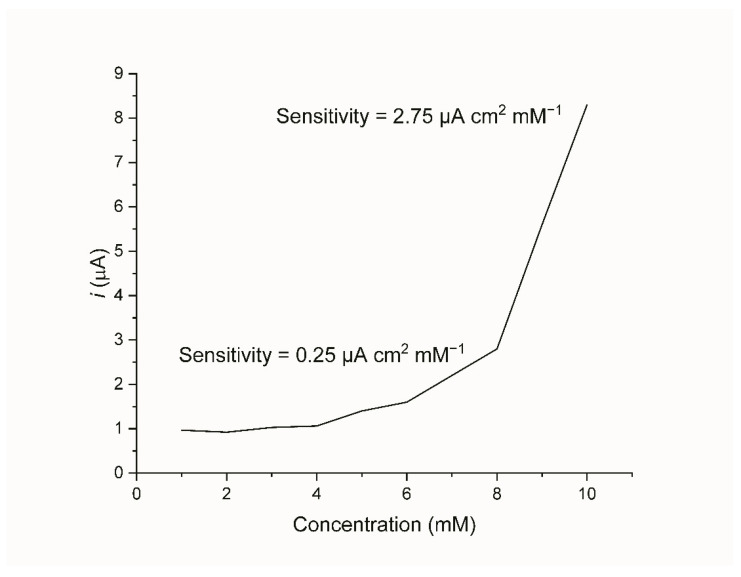
Calibration curve of the current verses different glucose concentrations (1 to 10 mM).

The Au-Ni/AC SPE has been tested for its specific response by the successive addition of glucose onto the sensor that was dipped in NaOH. The chosen range was 1–10 mM as the target concentration for the detection of yellow rust was 5 mM as based on the designed second layer. The calibration curve (Figure 11) shows that there was an exponential increase in the peak generated current with the addition of glucose, which is in agreement with the trends that can be found in the literature [52,53].

It can be observed that there are two linear ranges between 1–8 mM and another between 8–10 mM. Based on these ranges, the two sensitivities have been calculated using a linear fit are 0.25 μA cm^2^ mM^−1^ and 2.75 μA cm^2^ mM^−1^, respectively. The limit of detection (LOD) has also been tested experimentally to find the lowest concentration that the sensor can detect which was found to be 0.05 μM. The performance of this biosensor is in line with other non-enzymatic nanoparticle-based glucose biosensors in terms of its sensitivity, linear ranges, and LOD. The reported sensitivities are between $1\times 10^{-2}–1.04\;{\mu A\;\mathrm{cm}}^{2}{\;\mathrm{mM}}^{-1}$, while the linear ranges are between $7\times 10^{-5}–20$, and the LOD is 0.1–15 μM [54,55,56].

**Figure 12:**
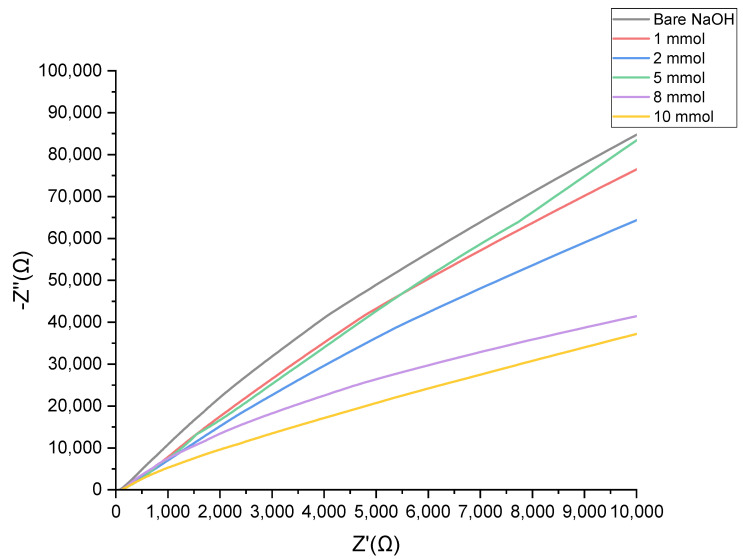
Nyquist plots for Au-Ni/AC SPE in 0.1 M NaOH solution for 0–10 mM glucose concentration range.

An electrochemical impedance spectroscopy was conducted to analyze the impedance changes on the modified electrode surfaces. In the EIS, the presence of a straight line represents that the diffusion limited the electrochemical process, which is the hypothesis of this paper [57]. Pletcher [58] suggests the electrocatalytic process implies that there is a key role for the adsorbed intermediates. Suggesting that the initial oxidation of the glucose was limited to the surface would result in a bulk oxidation which can be indicated by the linear behaviour that is presented in Figure 12.

**Figure 13:**
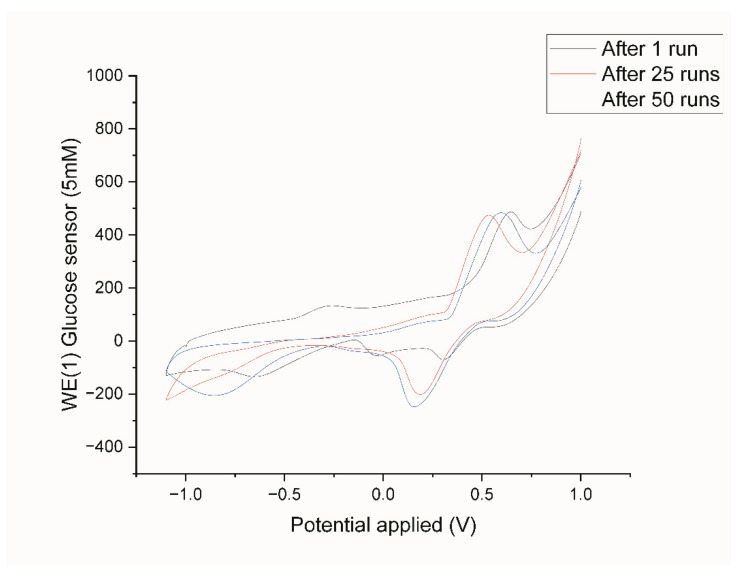
CVs of Au-Ni/AC SPE after 1, 25, and 50 times of amperometric tests in 5 mM glucose in 0.1 M NaOH.

The stability of the Au-Ni/AC SPE was tested by us performing cyclic voltammetry measurements before and after the amperometric analysis fifty times over, and these are shown in Figure 13. The results reveal that after a long period of electrolysis using the electrode, the current kept almost 100% of its initial amount, whilst the peak potential moved in a slightly positive direction by 0.6 V. The reproducibility and repeatability of the electrode were also determined by repeated testing, which was conducted for 1, 25, and 50 cycles in 5 mM glucose in 0.1 M NaOH. These results imply that the proposed electrode has a reproducible current as it was not contaminated by the oxidation products and the shift remained constant between the 1, 25, and 50 cycles.

### 3.3. Nanoparticles Characterisation

**Figure 14:**
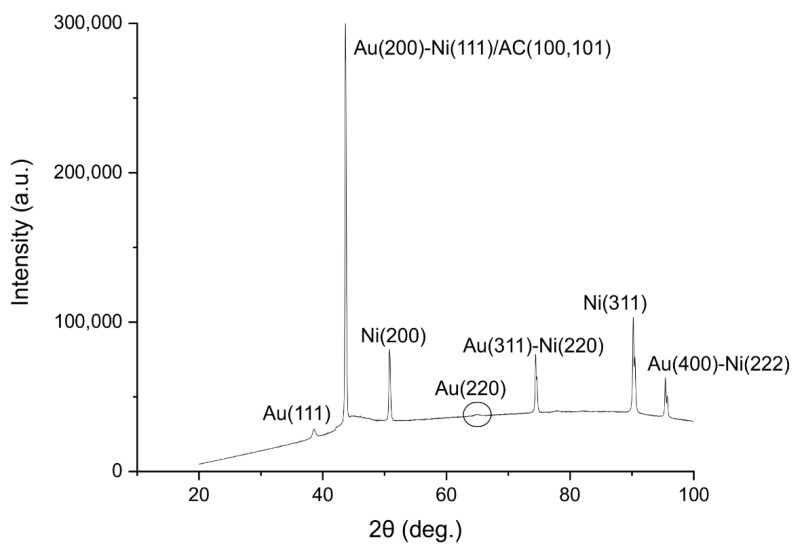
Au-Ni/AC X-ray diffraction analysis. The peaks represent the different materials present on the surface of the electrode. Au(111) at 38.6°, Au(200)-Ni(111)/AC(100)(101) at 43.7°, Ni(200) at 50.8°, Au(311)-Ni(220) at 74.4°, Ni(311) at 90.2°, and Au(400)-Ni(222) at 95.4°.

The XRD patterns of the Au-Ni/AC nanoparticles are presented in Figure 14, which show that there were characteristic diffraction peaks at 2θ = 38.6°, 43.7°, 50.8°, 64.9°, 74.4°, 90.2°, and 95.4°. As previously reported, the peaks confirm the presence of the elements Au and Ni: 38.7° Au (111), 43.7° Au(200)-Ni(111)/AC(100)(101), 50.8° Ni(200), 64.9° Au(220), 74.4° Au(311)-Ni(220), 90.2° Ni(311), and 95.4° Au(400)-Ni(222). Furthermore, the shift in the peaks indicates that the Au-Ni/AC alloy was present on the sensor surface [59,60]. Furthermore, the average crystallite diameter (*d*) values that were calculated using Scherrer’s equation [Equation (1)] are ∼41.8 nm for the Au-Ni/AC nanoparticles using the full width at half maximum (FWHM) of the most intense peak at 43.7°. β is the FWHM value (radians), ‘λ’ is 1.5406 Å, which is the CuKα wavelength, and θ is the Bragg diffraction [61,62].

D = 0.9λ/(β cos θ)(4)

### 3.4. Sensor Selectivity

The sensor was designed to operate at a temperature that ranged between 7 and 22 °C. A commercial screen-printed electrode was used to enable the connection to any CV machine, thereby allowing for its further integration in any system. Currently common wheat diseases include blotches, rusts, and head blight/scab [63]. Based on a genome analysis of all the agents that cause these diseases, it can be deduced that invertase production is a unique characteristic of *P. s.* f. sp. *tritici spores*, as is shown in Table 2 [26]. Hence, the sensor was designed to selectively detect the yellow rust pathogen.

### 3.5. Sensor Functionality

**Figure 15:**
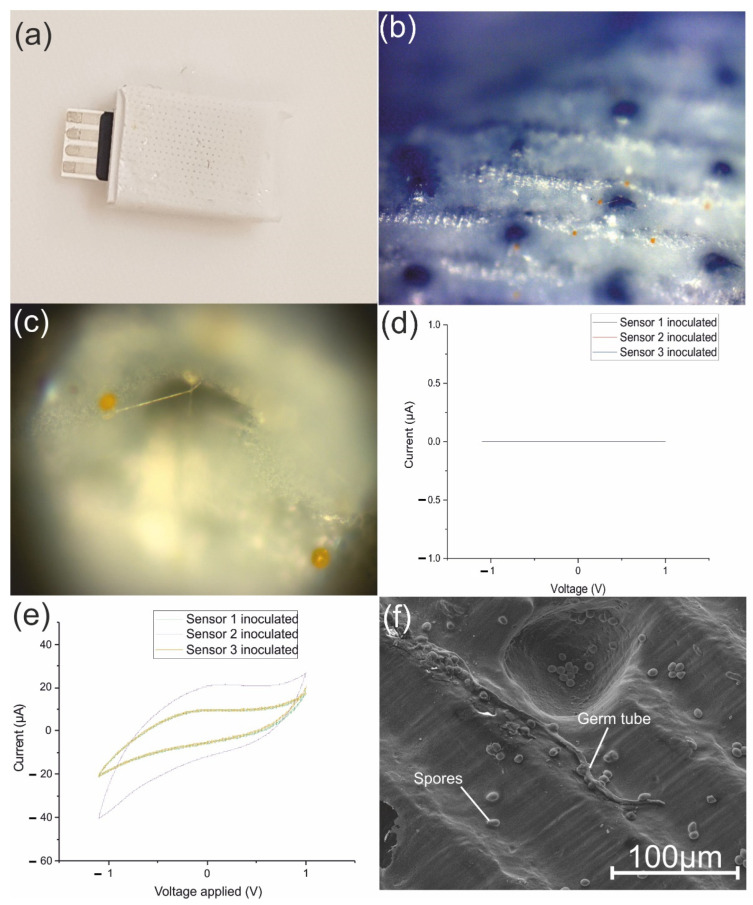
(**a**) Biosensor assembly, (**b**) top surface of biosensor inoculated with *P. s*. f. sp. *tritici* spores, (**c**) underneath surface of the top layer of the biosensor showing a germinated *P. s.* f. sp. *tritici* spore, (**d**) CV curve obtained at 24 h, (**e**) CV curve obtained after 72 h, (**f**) SEM image of *P. s.* f. sp. *tritici* spores and a germ tube growth on the surface of the biosensor.

After printing the PETG sensor body, the agar/sucrose layer was placed inside of the sensor body and covered with the casted mimetic layer. The assembled sensor (Figure 15a) was inoculated with spores of the yellow rust pathogen *P. s.* f. sp. *tritici* and placed in a temperature-controlled incubator at 7 °C with a humidity of 60% in total darkness for 24 h [70]. *P. s.* f. sp. *tritici* spore germination on the surface of the biosensor was assessed using a light microscopy method (Figure 15b,c). The sensor was connected to the CV system to test for the presence of glucose, which returned negative results at 24 h after inoculation (Figure 15d). The sensor was placed back in the incubator at a cycle of 16 °C/ 16 h and 7 °C/8 h, and it was tested for the presence of glucose again at 48 and 72 h after inoculation. At 48 h, the response was negative, but at 72 h a positive glucose response was obtained (Figure 15e). The biosensor was tested three times, thus confirming the results. The growth of *P. s.* f. sp. *tritici* on the surface of the biosensor was further investigated using an SEM method (Figure 15f).

## 4. Conclusions

A novel, multi-layer electrochemical biosensor is proposed for the detection of the fungal pathogen that causes the plant disease yellow rust of wheat, i.e., *P. s.* f. sp. *tritici*. The first layer of this biosensor mimics the wheat leaf surface morphology by containing artificial stomata. The second layer consists of a sucrose/VOC/agar mixture that act as a substrate, while a wheat terpenoid VOC provides a stimulus for *P. s.* f. sp. *tritici* germination and the germ tube growth. The third layer is fabricated by casting cheap and affordable nanomaterials; the formed structure on the surface presents an innovative nonenzymatic sensor that detects the glucose that is released from sucrose due to fungal invertase activity.

The inoculation of the biosensor with the spores of *P. s.* f. sp. *tritici* resulted in a positive reaction 72 h after the spore inoculation. This biosensor would therefore enable the pre-symptomatic yellow rust detection using an electrochemical approach. This proof of concept provides a substantial improvement to the current state-of-the-art biosensors, thereby reducing the detection cost and time that it takes. Therefore, the biosensor is a promising solution for food security, and it can prevent substantial annual crop yield losses that are due to *P. s.* f. sp. *Tritici*.

The proposed multilayer biosensor consists of three modular layers that can be easily changed to allow for the detection of different pathogens. The modularity can be achieved by changing the morphology of the first layer to mimic the target crop, by changing the feeding media (second layer) that is tailored towards the target pathogen, with the third layer being changed to detect the target analyte to signal the presence of the targeted pathogen. Moreover, the biosensor can be integrated as a part of a wider sensing network using the internet of things (IOT), thereby leading to the development of an early disease detection and warning system. Further work is required to optimise the nonenzymatic glucose sensor that is presented in this work. Different ratios of Au to Ni need to be investigated as well as different methods of immobilising the nanomaterial on the working area of the screen-printed electrode. Finally, the general electrochemical properties of the proposed system needs to be investigated and studied providing an in depth understanding of the mechanisms of the sensor’s functions.

## Figures and Tables

**Table 1:** Printing parameters.

Parameter	Value
Material	Formlabs Clear resin
Exposure time	1.7 s
Material Volume	3 mL
Number of layers	782
Layer height	1.7 µm

**Table 2:** List of common wheat diseases and their agents, indicating invertase production based on their genomic make-up.

Disease (Agent)	Invertase Production	Reference
Black rust (*Puccinia graminis*)	No	[64]
Yellow rust (*Puccinia striiformis*)	Yes	[26]
Leaf rust (*Puccinia triticina*)	No	[65]
Septoria blotch (*Zymoseptoria tritici*)	No	[66]
Septoria nodorum blotch (*Parastagonospora nodorum*)	No	[67]
Tan spot (*Pyrenophora tritici*)	No	[68]
Head blight/scab (*Fusarium graminearum*)	No	[69]

## Data Availability

Not applicable.
